# Within-subject assessment of swallowing threshold and efficiency for maxillary implant assisted overdentures with and without palatal coverage

**DOI:** 10.1007/s00784-025-06271-y

**Published:** 2025-04-15

**Authors:** Abdallah Mohammed Ibrahim, Elsayed Abdallah Abdel-Khalek, Osama Mohammed Askar

**Affiliations:** Department of Removable prosthodontics, Faculty of Dentistry, Mansoura University, Eldakahlia, 35516 Egypt

**Keywords:** Palatless, Maxillary, Implant overdenture, Threshold, Swallowing efficiency

## Abstract

**Objectives:**

Controversy remains regarding the impact of palatal coverage or palatless designs for maxillary implant overdentures. This within subject study aimed to compare the swallowing threshold and efficiency of maxillary implant assisted overdentures with and without palatal coverage.

**Materials and methods:**

The study included 14 healthy completely edentulous subjects with ages ranged from 50 to 70 years. For each participants, four dental implants were inserted in the canines and premolars areas of the maxillary arch, and two implants in the canines region of the mandibular arch. Two designs of maxillary implant-assisted overdentures (with and without palatal covreage designs) and one design of mandibular implant overdenture were constructed for each patient. The overdentures were retained to the implants by using locator attachments. Swallowing threshold and efficiency were evaluated using Test of Mastication and Swallowing Solids (TOMASS). Swallowing evaluation was conducted 3 months after each maxillary implant overdenture design insertion, while the participants were eating solid food. Independent samples t-test was used to compare the differences between the two overdenture designs.

**Results:**

There was statistically significant reduction in swallowing threshold, number of swallows, number of masticatory cycles, and total ingestion time for the maxillary implant overdenture without palatal coverage (*p* < 0.05). However, there was non-significant difference in the number of bites between the two maxillary implant overdenture designs (*p* > 0.05).

**Conclusion:**

Within the limitation of this study, it could be concluded that maxillary implant overdenture without palatal coverage improves chewing and swallowing efficiency for solid food.

**Clinical relevance:**

Palatal coverage with maxillary implant overdenture negatively affected bolus formation during oral food processing. Increased chewing cycles and time of mastication lead to increase the viscosity of the bolus, which might cause aspiration during swallowing. Maxillary implant overdenture without palatal coverage may be a viable treatment option as it improve the oral food processing for safe swallowing. In addition, TOMASS test is easy to be incorporated as part of the clinical evaluation of swallowing efficiency for solid foods processing.

## Introduction

Complete edentulism is more prevalent and occurs earlier in the maxillary arch compared to the mandibular arch [1, 2]. Patients who are treated with conventional complete dentures often showed some negative side effects related to resorption of the residual ridge that can affect mastication process and unhealthy selection of food types. Also, poorer oral health related quality of life due to chewing dysfunction [3]. The introduction of dental implants can prevent residual ridge bone loss and improve quality of life, and chewing ability [4, 5], making implant-assisted overdentures a reliable treatment modality for edentulous patients in both the mandibular and maxillary jaws [6–9].

Controversy remains regarding the impact of palatal coverage or without palatal coverage designs for maxillary implant overdentures (MaxIODs). From a mechanical standpoint, MaxIODs with palatal coverage have been shown to have superior mechanical properties compared to without palatal coverage designs [10, 11]. The attachment system wear and liability of denture base fracture can be reduced by using MaxIODs with palatal coverage. Additionally, prosthetic maintenance is less common with palatal coverage compared to without palatal coverage designs, especially without reinforcement [12]. However, the MaxIODs without palatal coverage design offers the benefit of uncovering the palatal area, which includes sensory innervation and minor salivary glands. This can lead to improve taste sensation, speech function, and reduce the overdenture bulk [2, 13].

Previous research studied the impact of palatal coverage on retention property of MaxIODs. The results showed that reducing palatal coverage did not have any effect on the retention of the MaxIODs during feeding. Additionally, the reduction of palatal coverage did not significantly influence the ability to withstand tilting loads [6].

In a 2-year clinical study, it was found that speech function did not improve without palatal coverage when compared to a conventional maxillary denture at the time of insertion of a maxillary implant assisted overdenture. Furthermore, there was no significant difference in speech function between the first and second year regardless of whether the overdenture had reduced or full palatal coverage [13]. Additionally, another study that evaluated the oral health impact profile found that non-significant difference was presented between MaxIODs with full and reduced palatal coverage except for esthetic and taste sensation [14].

Studies have yielded conflicting findings regarding the impact of palatal coverage on oral stereognosis. Some studies have suggested that the tongue is the primary mediator responsible for oral stereognosis and that the palatal area is not a significant factor [15, 16], they illustrated that the use of a maxillary denture may lead to an improvement in oral stereognosis. This is because the denture acts as a rigid structure against which the tongue can apply pressure while examining objects [16]. On the other hand, other studies suggest that the palatal area may play a role in oral stereognosis by altering the feedback from palatal mucosa receptors and changing the oral environment [17–20]. Furthermore, increasing complete denture retention and stability by placement of dental implants [16], resulting in improved oral awareness for denture wearers [21, 22].

Swallowing process includes 3 principle phases: oral, pharyngeal, and esophageal phases. Only, The oral phase is under voluntary control and involves the use of the lips, tongue, teeth, and masticatory muscles [23, 24]. Before the swallowing process can occur, the food should be chewed, and collected into a food bolus. The teeth, tongue, jaw coordination, facial muscles, and tongue-palate contact carry out this oral processing [25–27]. Swallowing function usually assessed using videofluoroscopy or videoendoscopy. These techniques require specialized instruments not typically found in clinics [28]. As a result, the “Test of Mastication and Swallowing Solids” (TOMASS) was presented to evaluate the oral and pharyngeal swallowing efficiency while consuming solid foods [29]. Numerous studies demonstrated significant psychometric properties for the TOMASS test since its development [30–32].

Previous published studies have shown that palatal coverage can have negative effects on swallowing function [33–35]. However, these studies have primarily focused on mastication and swallowing as separate functions. In other words, they have evaluated masticatory function without considering its impact on subsequent swallowing function. Additionally, there is limited information available on the effects of reduced palatal coverage on food processing during chewing and subsequent swallowing functions for edentulous patients. Therefore, the purpose of the study was to evaluate the swallowing threshold and efficiency of MaxIODs with and without palatal coverage, using TOMASS test. The null hypothesis of this study was no significant difference between the two MaxIODs designs.

## Materials and methods

### Patient selection and study design

This within subject study conducted on fourteen completely edentulous subjects seeking prosthetic treatment at the Prosthodontics Department, Faculty of Dentistry, Mansoura University. The study has been applied with the Code of Ethics of the World Medical Association, following the ethics stated in the Declaration of Helsinki. The ethical committee of the Faculty of Dentistry (A0408024RP) approved the study design and the study was registered to clinicalTrials.gov (NCT06622837). All subjects were aged between 50 and 70 years, had sufficient bone quantity, sufficient inter-arch space, normal maxilla-mandibular relation, and have been edentulous for at least one year. Patients with uncontrolled systemic diseases, parafunctional habits, temporo-mandibular joint disorders, and a history of head/neck surgery were excluded, as were smoking and those who undergo radiation therapy. The sample size was determined based on the number of chews for TOMASS test data from previous research [36]. A computer software program (G*power, version 3.1.5, Kiel, Germany) was used for sample size calculation. 14 participants was detected with an effect size of 72%, 5% error probability, and 80% power. The research protocol including the merits and demerits were explained to the participants and written consent were obtained from all patients. The study was conducted following CONSORT guidelines for clinical trials.

### Prosthetic and surgical procedures

First, new complete dentures were constructed for each participants using the conventional technique. The dentures were duplicated using clear acrylic resin material. Radiopaque gutta percha markers were placed on the labial, buccal, and lingual flanges of the clear resin duplicate at different axial planes to create a radiographic stent.

For each participant, dual scan was performed using cone beam computerized tomography (i-CAT, Pennsylvania, USA). One scan was taken while the patient wore the radiographic stent, and the other scan was taken for the stent extra orally. These two scans were then superimposed to create an implant plan and construct stereolithographic surgical guides. With the assistance of these stereolithographic surgical guides, the universal surgical guide with sequential surgical drilling was used to insert 4 dental implants in the canines and premolars regions of the maxillary arch (3.5 mm width and 11.5 mm length at the canine areas and 10 mm length at the second premolar). In addition, 2 dental implants (3.5 mm width and 13 mm length) were placed in the canines region of the mandibular arch (IS III Active platform Implant, Neo BioTech). Submerged surgical technique and delayed loading protocol were used.

Each participants instructed to take Prophylactic broad spectrum antibiotic (875 mg Amoxicillin and Clavulanic acid 125 mg) one hour before the surgery and for 7 days and rinsed their mouth with an antiseptic mouthwash (2% chlorohexidine) for 2 weeks after surgery. Additionally, non-steroidal anti-inflammatory drug (Diclofenac tablet 50 mg) was prescribed to control postoperative pain. One week after the surgery, the dentures were relived and relined with soft liner material (Mucosoft, Parkell. USA) and delivered to the participants. Follow-up visits were scheduled within the osseointegration period (3 months for the mandibular arch and 6 months for the maxillary arch).

**Fig. 1:**
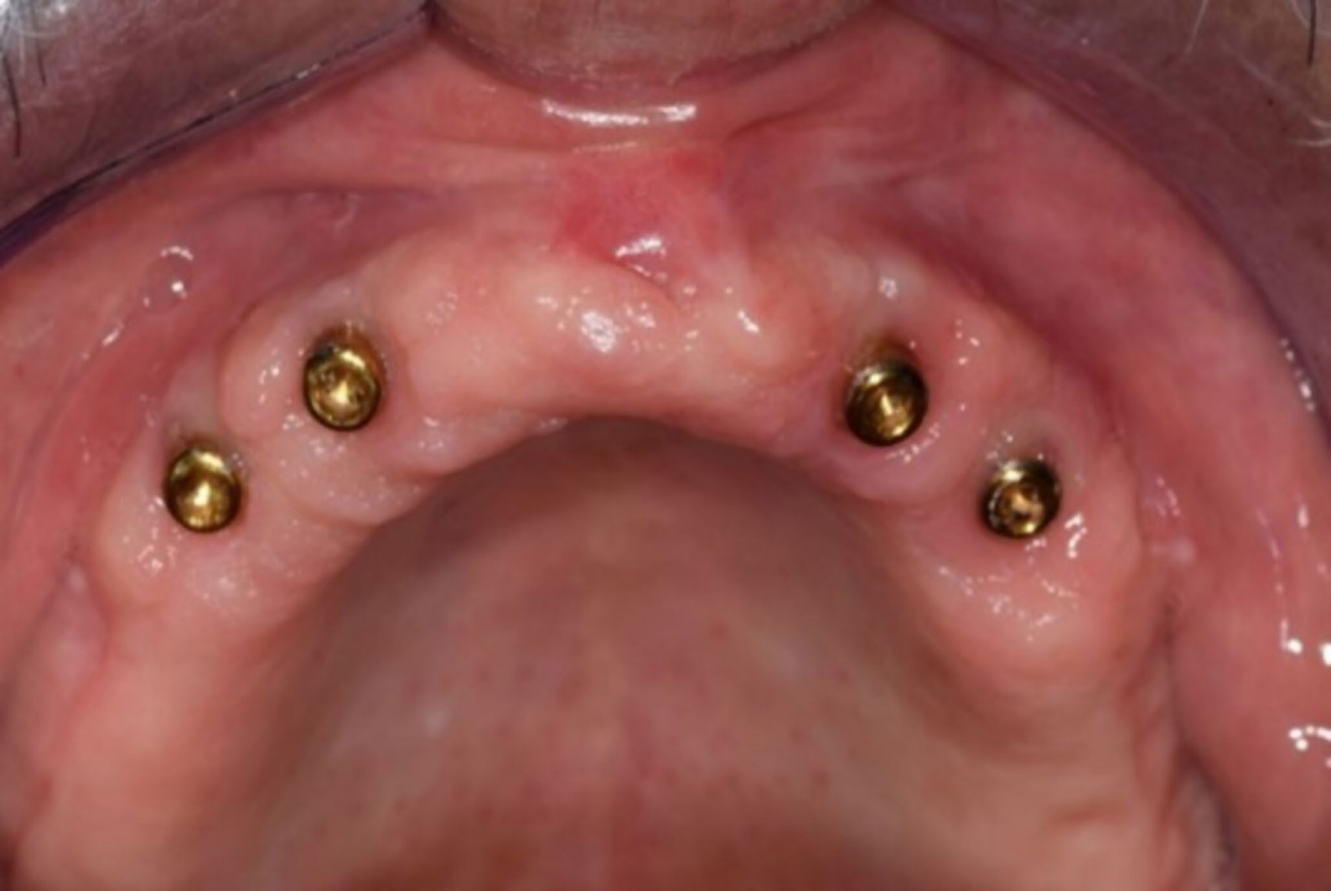
Intra oral view of locator abutments

**Fig. 2:**
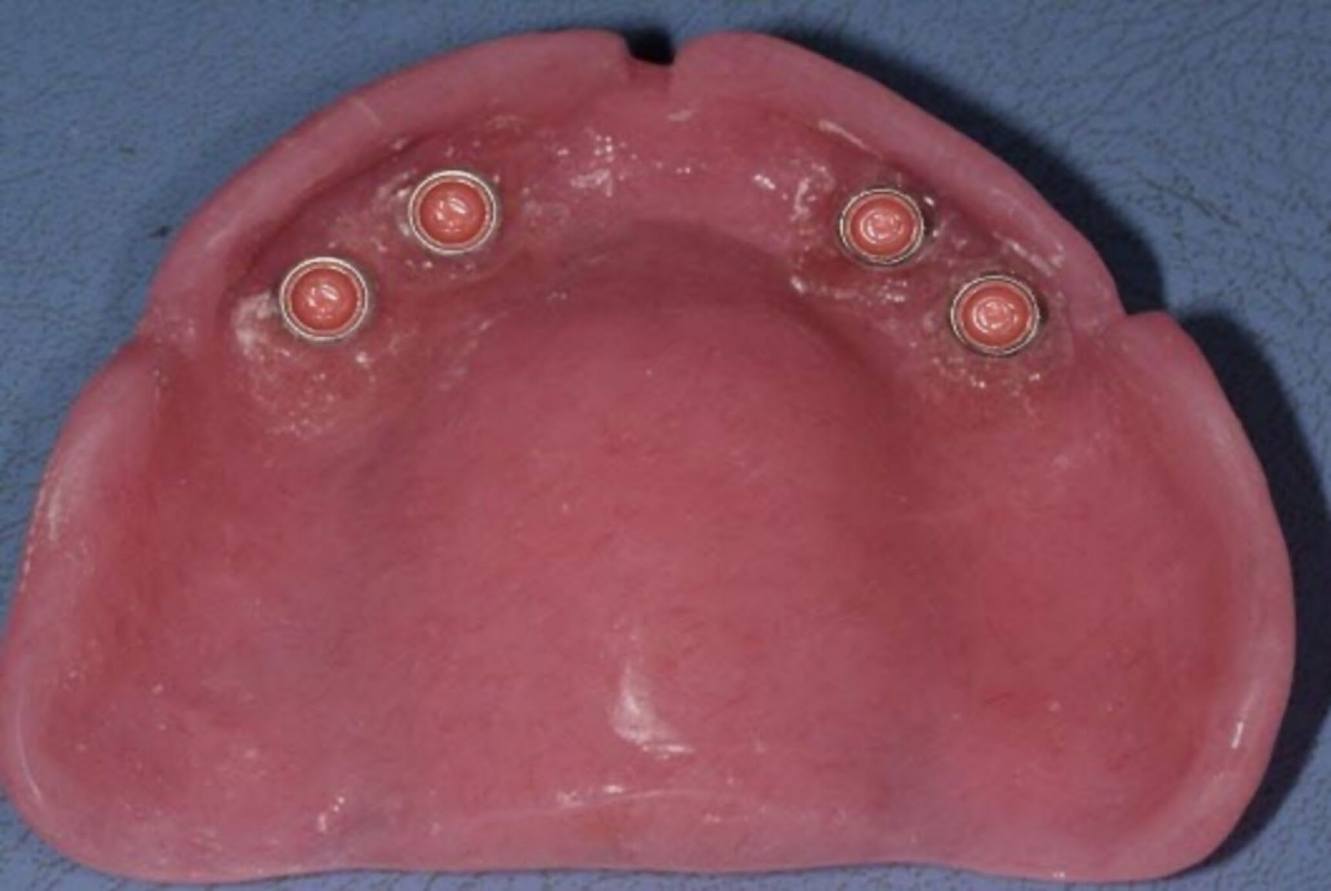
Maxillary implant overdenture with palatal coverage fitting surface with the attachment housings

**Fig. 3:**
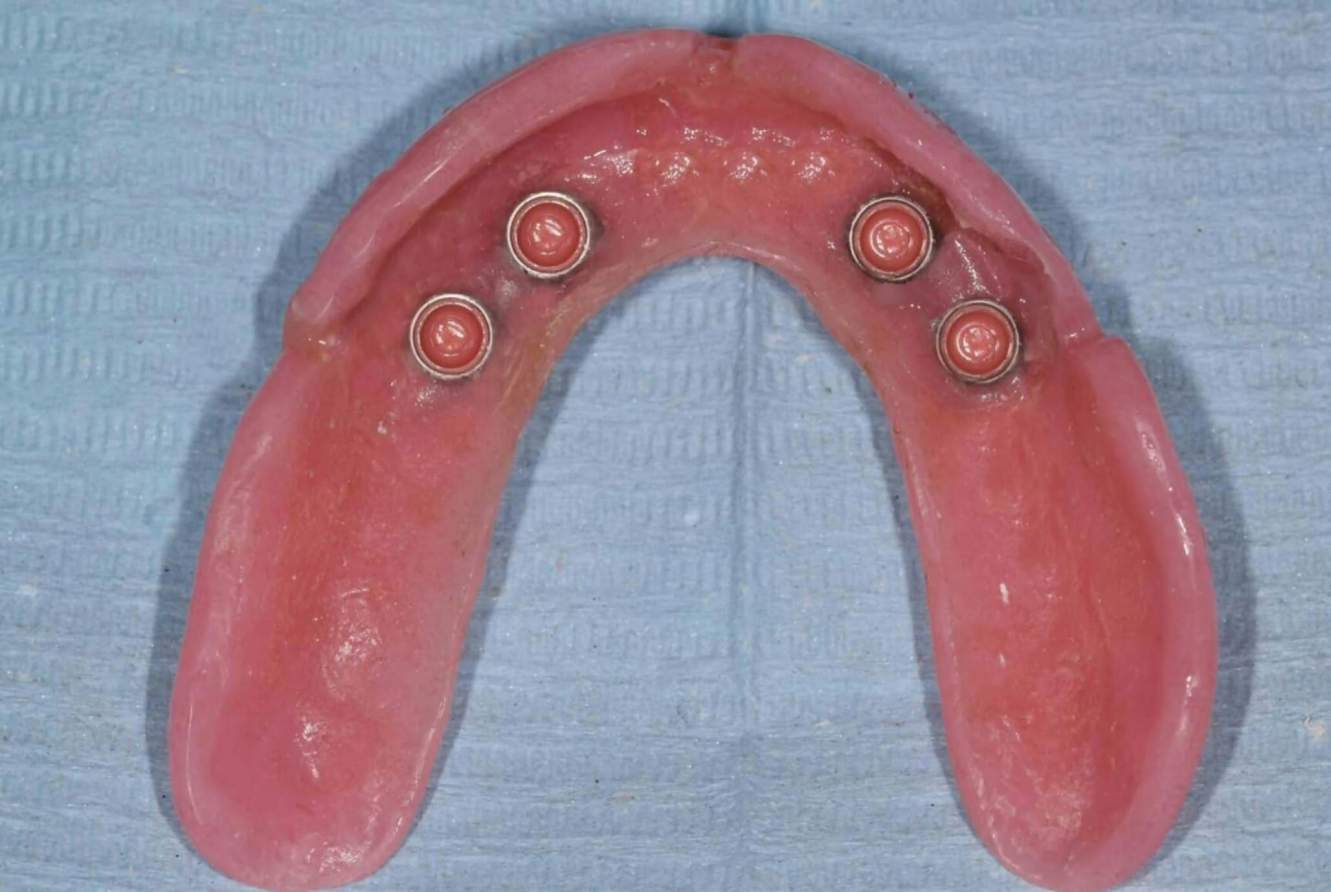
Maxillary implant overdenture without palatal coverrage fitting surface with the attachment housings

After the osseointegration period, locator attachments (3 mm cuff height, Neo Bio Tech Co., Ltd, Korea) were screwed to the implants (Fig. 1). Each patient received the following types of overdentures:

1. One mandibular implant-retained overdenture: After reliving the mandibular denture, chair-side technique was used to incorporate the attachments housing into the fitting surface of the mandibular denture.
2. Two designs of MaxIODs:
  MaxIOD with palatal coverage: After relieving the conventional maxillary denture, chair-side technique was used to incorporate the attachments housing into the fitting surface of the conventional maxillary denture (Fig. 2).
  MaxIOD without palatal coverage: The conventional maxillary denture was duplicated and the palatal part was cut out, finished, and polished. The fitting surface was relived and chair-side technique was used to incorporate the attachments housing into the fitting surface of the maxillary denture without palatal coverage (Fig. 3).

The participants were randomly allocated into two equal groups (7 per group). An independent examiner using a computer program, specifically Microsoft Excel, carried out randomization process. The first group of 7 participants received MaxIODs with palatal coverage. After a period of three months, the TOMASS test was done. Following this, they wore a MaxIODs without palatal coverage for an additional 3 months, after which the test was repeated. The second group of 7 patients initially received MaxIODs without palatal coverage. After three months of use, the TOMASS test was done. They then received MaxIODs with palatal coverage and the test was repeated after an additional three months.

### Swallowing assessment

TOMASS test was utilized to assess participants’ swallowing abilities [30]. For this study, a commercially available cracker (biscuit) was used. Participants were instructed to sit on a chair in upright position and to eat the biscuit comfortably and as quickly as possible. They were also asked to say their name aloud as it indicate that they had finished the test. Using a video recorder the procedure was recorded for further analysis.

The recorded videos were then analyzed using the Avidemux 2.6 program to measure the following:

1. Swallowing threshold, which is the duration of chewing until the first swallow (determined by the moment of laryngeal elevation).
2. The TOMASS test parameters:
  - Number of bites: The number of discrete bites taken to finish the cracker.
  - Number of masticatory cycles: As each cyclical mandibular movement up–down.
  - Number of swallows: As each vertical movement of the thyroid cartilage.
  - Total ingestion time: The time taken to complete the ingestion of the cracker from the first bite to the last.

All measurements were conducted by the same investigator and repeated 3 times and the mean was used in the analysis. At the end of evaluation, the patients were asked to choose, which types of MaxIODs they would like to retain.

### Statistical analysis

The data were analyzed using statistical package for social science version 20 (SPSS Inc.). Shapiro-Wilk test was used to assess the normal distribution of data. The data were parametric and met the normal distribution. Consequently, descriptive statistics were presented using mean, and standard deviations. Comparison of swallowing threshold and TOMASS parameters between MaxIODs with and without palatal coverage was made by Independent samples t-test. *P* < 0.05 was considered to represent statistically significant difference.

## Results

The base line of the participants enrolled in this study was presented in Table 1. The mean age was (60.5 ± 6.1 years) and 10 participants were males while 4 participants were females. The mean duration of edentulism was (3.78 ± 1.47 years). About educational status, 2 participant (14.3%) had high education, 5 participants (35.7%) had medium education, and 7 participants (50%) had low education.

The mean values for swallowing threshold and TOMASS test parameters were illustrated in Table 2 for MaxIODs with and without palatal coverage. The swallowing threshold, number of bites, number of masticatory cycles, number of swallows, and total time were higher in maxillary implant overdenture with palatal coverage (14 ± 1.8, 3.7 ± 1.1, 61.9 ± 12.8, 3.5 ± 0.6, 54.1 ± 6.7, respectively ) compared to without palatal coverage design (11.6 ± 1.4, 3.1 ± 0.8, 49.6 ± 6.1, 2.7 ± 0.7, 41.6 ± 5.5, respectively).

Statistically significant reduction in swallowing threshold was found for MaxIODs without palatal coverage when compared to MaxIODs with palatal coverage (*p* < 0.05). For TOMASS test parameters, there was statistically significant reduction in number of masticatory cycles, number of swallows, and Total time for MaxIODs without palatal coverage compared to MaxIODs with palatal coverage (*p* < 0.05). While, non-significant difference was found for number of bite between the two MaxIODs designs (*p* > 0.05).

At the end of evaluation period almost of subject (12 patients 85.7%) selected to keep the MaxIODs without palatal coverage.

**Table 1:** Baseline information of the participants (*n* = 14)

Characteristics	No. (%)
**Age (years)**,** mean ± SD**	60.5 ± 6.1
**Gender (male/ female)**	10:4
**Duration of edentulism (years)**,** mean ± SD**	3.78 ± 1.47
**Educational status**:	
- **Low** - **Medium** - **High**	7 (50%) 5 (35.7%) 2 (14.3%)

SD: Standard deviation

**Table 2:** Comparison of swallowing threshold and test of mastication and swallowing solids parameters for maxillary implant assisted overdentures with and without palatal coverage

Variables		MaxIODs with palatal coverage	MaxIODs without palatal coverage	*P* value (independent sample t-test)
		Mean ± SD	Mean ± SD	
**Swallowing threshold (sec.)**		14 ± 1.8	11.6 ± 1.4	0.02*
**TOMASS**	**Number of bites**	3.7 ± 1.1	3.1 ± 0.8	0.07
	**Number of masticatory cycles**	61.9 ± 12.8	49.6 ± 6.1	0.004*
	**Number of swallows**	3.5 ± 0.6	2.7 ± 0.7	0.002*
	**Total time (sec.)**	54.1 ± 6.7	41.6 ± 5.5	0.003*

MaxIODs: Maxillary implant assisted overdentures

TOMASS: Test of mastication and swallowing solids

SD: Standard deviation *p is significant at 5%

## Discussion

Eating capability is an important factor to consider for individuals who have difficulty chewing or swallowing, such as those who wear dental prosthesis [37]. To ensure a smooth and safe swallowing experience, it is crucial to create a bolus that is easy to swallow through proper mastication [38]. MaxIODs have been shown to have a high success rate and are considered a safe and reliable treatment option for completely edentulous patients, especially when combined with mandibular overdentures [39, 40]. In fact, this study have shown that MaxIODs without palatal coverage can significantly improve swallowing threshold and efficiency compared to with palatal coverage design. As a result, the null hypothesis of the study was rejected.

The swallowing threshold was significantly decreased for MaxIODs without palatal coverage. This may be due to the MaxIODs with palatal coverage cover the palatal area, which may disrupt the tongue’s motor ability and reduce sensory-motor coordination during mastication. During the process of oral food processing, the tongue plays a crucial role in retrieving and repositioning food on the occlusal surfaces of the teeth through its anteroposterior, medio-lateral, and rotational movements [41, 42]. Similarly Kaiba et al., found that there was statistically significant reduction of masticatory efficiency after the placement of an experimental palatal plate [17]. Also Zembic et al., study found that taste sensation was significantly improved for maxillary implant overdenture without palatal coverage [14].

The findings of this study indicate that the number of masticatory cycles for solid food was significantly higher for patients with MaxIODs with palatal coverage compared to without palatal coverage design. This could be attributed to the fact that patients increased the number of mastication strokes in order to form a bolus that is easier to swallow. In a study by Sato et al., it was observed that dentulous subjects who used artificial palatal plates experienced a decline in indices of bolus formation after palatal plate insertion. However, after one week, the indices returned to normal but with an increase in masticatory cycles. This change is a compensatory mechanism to improve masticatory performance and create a more comfortable bolus for swallowing [38].

The prolonged time required for managing solid food with MaxIODs with palatal coverage may be due to the palatal mucosa contain deep receptors, which are crucial for both masticatory, and swallowing function [43]. The decrease in sensory perception in the palatal area caused by MaxIODs with palatal coverage, results in less control during the oral phase of swallowing and impairs the role of the palate in fixing tongue movement to generate the swallowing process [38]. This finding is in accordance with Salgado et al., they found significant differences in mean swallowing times between dentate individuals and those with complete dentures, as well as between dentate individuals and those with implant-supported overdentures [44]. Another possible explanation for the prolonged time required for ingestion of solid foods may be the reduced masticatory efficiency caused by covering the palatal area [35].

In this study, it was observed that all patients consumed more than two swallows in order to finish their solid food. According to the Okada et al., study, even with just one bite of food, humans require more than one swallow when eating freely. The first swallow typically contains the majority of the food, with the remaining food being swallowed in subsequent swallows [45]. The use of MaxIODs with palatal coverage was found to result in an increased number of swallows compared to without palatal coverage. This could be attributed to an increase in masticatory strokes, a decrease in masticatory efficiency, and an increase in masticatory duration due to the palatal area being covered [38].

Ill-fitting dentures have negative impacts on swallowing function [46]. So new complete dentures were constructed with optimum denture base extension and proper denture base adaptation were constructed. Also direct pickup technique was used to retain the overdenture to the dental implant, which allow intimate tissue contact of the denture base and prevent food accumulation beneath the denture.

Based on finding of Kodaira et al., [33] study, they found that palatal morphometric feature could affect the bolus propulsion time during swallowing. In addition, the TOMASS test may be affected by the age and gender of the participants [32]. Therefore, this within subject study design allows standardization of patient factors. However, the limitations of this study included small sample size and the lack of evaluation of the impact of assessment time on swallowing function for MaxIODs. Future research on the effect of different food textures should be also investigated.

## Conclusion

Based on these findings, it can be concluded that: Palatal coverage with MaxIODs negatively affect the bolus formation during mastication and swallowing of food. Chewing and swallowing efficiency were improved with MaxIODs without palatal coverage than with palatal coverage.

## Data Availability

No datasets were generated or analysed during the current study.

## Abbreviations

MaxIODs: Maxillary implant overdentures
TOMASS: Test of Mastication and Swallowing Solids
